# Off-Label Use of Tenecteplase for the Treatment of Acute Ischemic Stroke: A Systematic Review and Meta-analysis

**DOI:** 10.1001/jamanetworkopen.2022.4506

**Published:** 2022-03-31

**Authors:** Aristeidis H. Katsanos, Klearchos Psychogios, Guillaume Turc, Simona Sacco, Diana Aguiar de Sousa, Gian Marco De Marchis, Lina Palaiodimou, Dimitrios K. Filippou, Niaz Ahmed, Amrou Sarraj, Bijoy K. Menon, Georgios Tsivgoulis

**Affiliations:** 1 Division of Neurology, McMaster University/Population Health Research Institute, Hamilton, Ontario, Canada; 2 Acute Stroke Unit, Metropolitan Hospital, Piraeus, Greece; 3 Department of Neurology, GHU Paris Psychiatrie et Neurosciences, Hôpital Sainte-Anne, Paris, France; 4 Department of Neurology, Université de Paris, France; 5 Department of Neurology, INSERM U1266, Paris, France; 6 Department of Neurology, FHU Neurovasc, Paris, France; 7 Neuroscience Section, Department of Biotechnological and Applied Clinical Sciences, University of L'Aquila, L’Aquila, Italy; 8 Department of Neurosciences (Neurology), Hospital de Santa Maria, University of Lisbon, Lisbon, Portugal; 9 Neurology and Stroke Center, Department of Clinical Research, University Hospital of Basel, University of Basel, Basel, Switzerland; 10 Second Department of Neurology, Attikon Hospital, School of Medicine, National and Kapodistrian University of Athens, Athens, Greece; 11 Department of Anatomy and Surgical Anatomy, Medical School, National and Kapodistrian University of Athens, Athens, Greece; 12 National Organization for Medicines (EOF), Athens, Greece; 13 Department of Neurology, Karolinska University Hospital, Stockholm, Sweden; 14 Department of Clinical Neuroscience, Karolinska Institute, Stockholm, Sweden; 15 Department of Neurology, UT Houston, Houston, Texas; 16 Calgary Stroke Program, Department of Clinical Neurosciences, Radiology and Community Health Sciences, University of Calgary, Calgary, Alberta, Canada; 17 Department of Neurology, University of Tennessee Health Science Center, Memphis

## Abstract

**Importance:**

Tenecteplase is being evaluated as an alternative thrombolytic agent for the treatment of acute ischemic stroke (AIS) within ongoing randomized clinical trials (RCTs). In addition, nonrandomized clinical experiences with off-label use of tenecteplase vs alteplase for AIS treatment are being published.

**Objective:**

To evaluate the available evidence on the safety and efficacy of intravenous tenecteplase compared with intravenous alteplase provided by nonrandomized studies.

**Data Sources:**

Eligible studies were identified by searching MEDLINE and Scopus databases. No language or other restrictions were imposed. The literature search was conducted on October 12, 2021. This meta-analysis used the Preferred Reporting Items for Systematic Reviews and Meta-Analyses (PRISMA) guidelines and was written according to the Meta-analysis of Observational Studies in Epidemiology (MOOSE) proposal.

**Study Selection:**

Nonrandomized studies (prospective or retrospective) comparing intravenous tenecteplase (at any dose) with intravenous alteplase in patients with AIS were included in the analysis.

**Data Extraction and Synthesis:**

The crude odds ratios (ORs) and 95% CIs were calculated for the association of tenecteplase vs alteplase with the outcomes of interest and adjusted ORs were extracted if provided. Estimates using random-effects models were pooled.

**Main Outcomes and Measures:**

The primary outcome was the probability of good functional outcome (modified Rankin scale [mRS] score, 0-2) at 90 days.

**Results:**

Six studies were identified including a total of 1820 patients (618 [34%] treated with tenecteplase). Patients receiving tenecteplase had higher odds of 3-month good functional outcome (crude odds ratio [OR], 1.22; 95% CI, 0.90-1.66; adjusted OR, 1.60, 95% CI, 1.08-2.37), successful recanalization (crude OR, 2.82; 95% CI, 1.12-7.10; adjusted OR, 2.38; 95% CI, 1.18-4.81), and early neurological improvement (crude OR, 4.88; 95% CI, 2.03-11.71; adjusted OR, 7.60; 95% CI, 1.97-29.41). No significant differences were detected in 3-month excellent functional outcome proportions (mRS score 0-1; crude OR, 1.53; 95% CI, 0.81-2.91; adjusted OR, 2.51; 95% CI, 0.66- 9.49), symptomatic intracranial hemorrhage (crude OR, 0.97; 95% CI, 0.44-2.16; adjusted OR, 1.16; 95% CI, 0.13-10.50), or parenchymal hematoma (crude OR, 1.20; 95% CI, 0.24-5.95).

**Conclusions and Relevance:**

Evidence from nonrandomized studies suggests tenecteplase is as safe as alteplase and potentially associated with improved functional outcomes compared with alteplase. Based on these findings, enrollment in the ongoing RCTs appears to be appropriate.

## Key Points

**Question:**

How does the use of tenecteplase compare with the use of alteplase in the clinical outcomes of patients with acute ischemic stroke (AIS) receiving intravenous thrombolysis?

**Findings:**

In this systematic review and meta-analysis, 6 nonrandomized studies including 1820 participants were analyzed. Intravenous tenecteplase was associated with better short-term and long-term functional outcomes in patients with AIS and a higher likelihood of successful recanalization in patients with acute intracranial vessel occlusions; no increased risk of intracranial bleeding was noted with intravenous tenecteplase compared with alteplase.

**Meaning:**

Analysis of evidence from nonrandomized studies suggests that tenecteplase is as safe as alteplase for the treatment of AIS and tenecteplase is potentially associated with more favorable outcomes.

## Teaser

The systematic review and meta-analysis compares the use of tenecteplase vs alteplase in the treatment of patients with acute ischemic stroke.

## Introduction

Tenecteplase has a well-characterized mechanism of action with important practical advantages in administration and superior clinical efficacy for patients with large-vessel occlusion, as demonstrated by randomized clinical trials (RCTs).^1,2,3^ Despite the advantages of tenecteplase and the recent endorsement of its use in national and international guidelines,^4,5,6^ alteplase remains the only regulatory-approved intravenous thrombolytic agent for the treatment of acute ischemic stroke (AIS). Although the use of intravenous tenecteplase for acute stroke treatment is still considered off-label, intravenous tenecteplase is increasingly being used for the treatment of AIS, particularly in countries where tenecteplase has a lower cost than alteplase.^7,8,9^

Because several stroke centers around the world have published their local experience with the off-label use of intravenous tenecteplase for AIS, we decided to perform a systematic review and meta-analysis to evaluate the available evidence on the association of intravenous tenecteplase compared with intravenous alteplase with the outcomes provided by these nonrandomized studies.

## Methods

The systematic review and meta-analysis is reported according to the Preferred Reporting Items for Systematic Reviews and Meta-analyses (PRISMA) reporting guideline and adheres to the Meta-analysis of Observational Studies in Epidemiology (MOOSE) proposal.

Three of us (A.H.K., K.P., and G. Tsivgoulis) searched MEDLINE and Scopus databases for nonrandomized studies (prospective or retrospective) reporting outcomes of patients with AIS receiving intravenous thrombolysis with either tenecteplase or alteplase at any dose. The last literature search was performed on October 12, 2021. No language or other restrictions were applied in the literature search algorithm. Conference proceedings from the European Stroke Organization, American Stroke Association, and World Stroke Organization were also screened after the database literature search. Studies reporting only experience with intravenous tenecteplase treatment, without including a comparison intravenous alteplase control group, were excluded. In studies with overlapping participant data, we selected a single publication including the highest number of total participants. Case reports and case series were excluded from further consideration. Risk of bias for each eligible study was assessed with the Newcastle-Ottawa Scale by the 2 of us who performed the literature search (A.H.K. and K.P.).^10^ This scale uses multiple-choice questions to address the areas of selection, comparability, and exposure/outcome assessment. High-quality ratings are identified with a star and studies can earn a maximum of 9 star-points.^10^ All conflicts during the literature search and bias assessment were resolved after discussion.

The primary outcome of interest was a modified Rankin Scale (mRS) score of 0 to 2 at 3 months.^11^ Secondary efficacy outcomes of interest included successful recanalization in patients with confirmed intracranial vessel occlusion according to the definition used in each study (eTable 1 in the Supplement), early neurologic improvement according to the definition used in each study (eTable 2 in the Supplement), and excellent functional outcome, defined as 3-month mRS scores of 0 or 1.^11^ Primary safety outcome included symptomatic intracranial hemorrhage. Any parenchymal hematoma following intravenous thrombolysis treatment constituted the secondary safety end point.

### Statistical Analysis

For each outcome of interest, we extracted or calculated the crude odds ratios (ORs) and corresponding 95% CIs. All adjusted ORs and corresponding 95% CIs were extracted from each study. The adjustment for potential confounders in different studies is displayed in eTable 3 in the Supplement. Study estimates were pooled under the random-effects model. Heterogeneity between studies was assessed with the Cochran *Q* and *I*^2^ statistics. For the qualitative interpretation of heterogeneity, *I*^2^ values of at least 50% were considered to represent substantial heterogeneity, and values of at least 75% indicated considerable heterogeneity.^12^ Publication bias was evaluated graphically by inspection of a funnel plot for the primary outcome.^13^ The significance threshold was set at α = .05. All statistical analyses were conducted with RevMan, version 5.3 software (Cochrane Collaboration).

## Results

**Figure 1.**
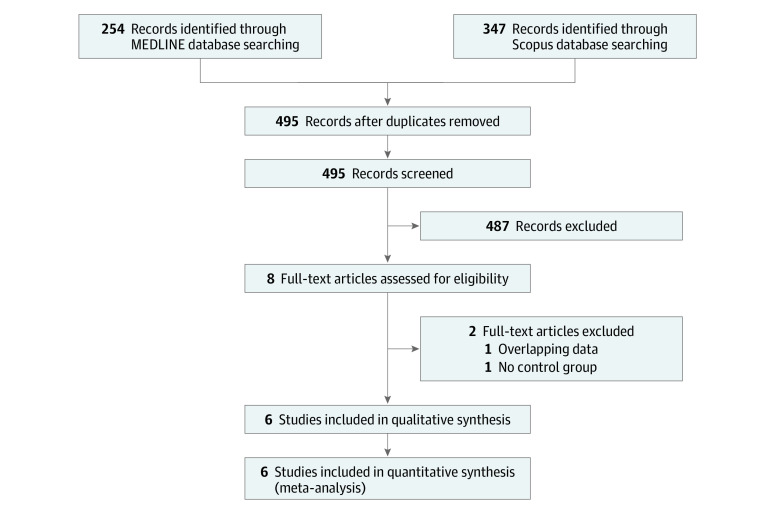
Selection of Eligible Studies

We analyzed aggregate data from 6 studies, including 1820 participants (618 [34%] treated with tenecteplase). A literature search retrieved 254 records from MEDLINE and 347 records from Scopus. After excluding duplicates, we identified 8 studies potentially eligible for inclusion. Two of these studies were excluded—one included overlapping participant data with another publication and the other included no intravenous alteplase treatment control group (Figure 1).^14,15^ Characteristics of the studies that were eligible for the meta-analysis are presented in Table 1.^16,17,18,19,20,21^

**Table 1.** Overview of Included Studies

Source (location)	Recruitment period	Study design	No. patients (% women)	Dose, mg/kg		Median age, y		Median NIHSS		Median (IQR) OTT, min	
				Tenecteplase	Alteplase	Tenecteplase	Alteplase	Tenecteplase	Alteplase	Tenecteplase	Alteplase
Alemseged et al,^16^ 2021 (multicenter)	October 2009-July 2019	Pooled analysis of patient level data from RCT and prospective registry data	110 (36)	0.25 or 0.4	0.9	77^a^	67^a^	20	15	160 (130-200)	181 (93-203)
Parsons et al,^17^ 2009 (Australia)	January 2006-July 2007	Pilot prospective, open-label, nonrandomized, controlled trial	50 (NA)	0.1	0.9	73^a^	69.4^a^	14	15	204 (24)^a^	13 8 (24)^a^
Psychogios et al,^18^ 2021 (Greece)	January 2016-March 2020	Pilot prospective, open-label, nonrandomized	58 (43)	0.25	0.9	69	70	19	16	165 (105-230)	165 (130-220)
Seners et al,^19^ 2019 (France)	May 2015-October 2017	Retrospective, propensity-score matching	262 (NA)	0.25	0.9	74	69	16	15	145 (123-175)	149 (120-180)
Mahawish et al,^20^ 2021 (New Zealand)	January 2018-February 2020	Retrospective observational	838 (47)	0.25 or 0.4	0.9	71.8^a^	71.9^a^	8	8	NA	NA
Warach et al,^21^ 2021 (US)	September 2017-December 2020	Prospective, observational, open-label, sequential cohort registry study	502 (NA)	0.25	0.9	66	67	8	8	NA	NA

Abbreviations: NA, not available; NIHSS, National Institutes of Health Stroke Scale; OTT, onset-to-treatment time; RCT, randomized clinical trial.

^a^ Mean value (SD).

In the quality control of included studies, we noted selection issues with cases (tenecteplase treatment) and controls (alteplase treatment) (eTable 4 in the Supplement). Alemseged et al^16^ included only patients with basilar artery occlusion treated with either intravenous tenecteplase or intravenous alteplase, thus limiting the generalizability of their findings. Data in the study by Alemseged et al^16^ were derived from a retrospective analysis of patients with basilar artery occlusion prospectively enrolled either in the Tenecteplase vs Alteplase Before Endovascular Therapy for Ischemic Stroke trial or the Basilar Artery Treatment and Management registry. In the study by Parsons et al,^17^ controls (alteplase treatment) were patients with AIS presenting within 3 hours from symptom onset and without certain neuroimaging criteria, whereas cases (tenecteplase treatment) represented patients with AIS presenting between 3 and 6 hours from symptom onset who fulfilled certain neuroimaging criteria. In the study by Seners et al,^19^ the control group comprised hospitalized patients with AIS who received alteplase treatment at different institutions. In the studies by Psychogios et al^18^ and Mahawish et al,^20^ data on patients treated with intravenous tenecteplase were prospectively collected; however, those who received intravenous alteplase were from historical cohorts at these same institutions. Regarding outcome assessment, 3 of 6 studies reported no blinding of outcome adjudicators to treatment.^18,19,20^ In addition, one of these studies^20^ reported a substantial proportion of patients lost to 3-month follow-up with imbalance in lost-to-follow-up rates between patients treated with intravenous alteplase (50 [18%]) or intravenous tenecteplase (77 [14%]).The full text of 1 of the studies had not been published at the time of our systematic review; thus, bias assessment was not possible.^21^

**Figure 2.**
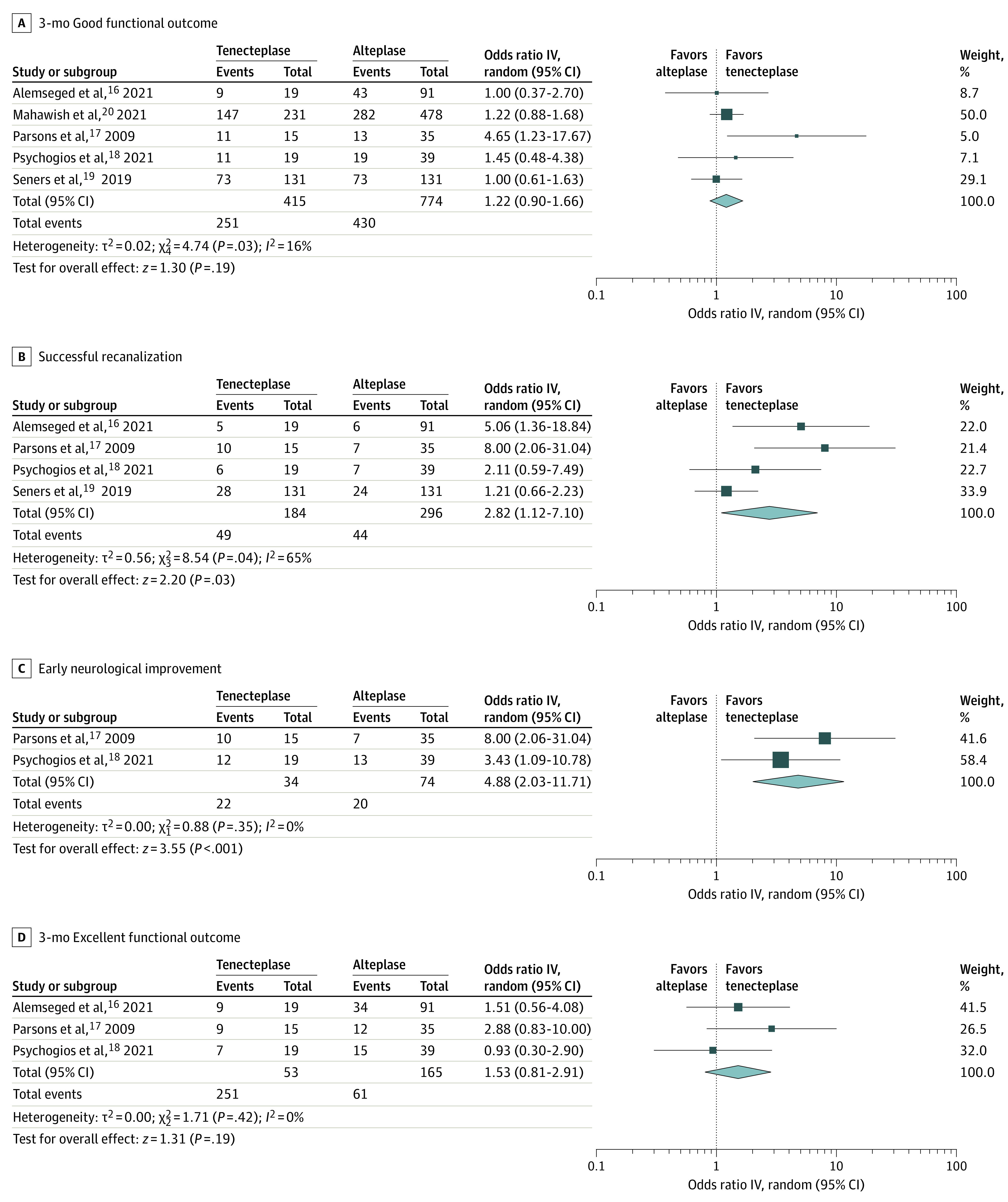
Unadjusted Analyses on the Comparison Between Intravenous Tenecteplase and Alteplase Outcomes of (A) 3-month good functional outcome (modified Rankin scale 0-2), (B) successful recanalization, (C) early neurological improvement, and (D) 3-month excellent functional outcome (modified Rankin Scale 0-1). The size of squares is proportional to the weight of each study. Horizontal lines indicate the 95% CI of each study; diamond, the pooled estimate with 95% CI.

**Figure 3.**
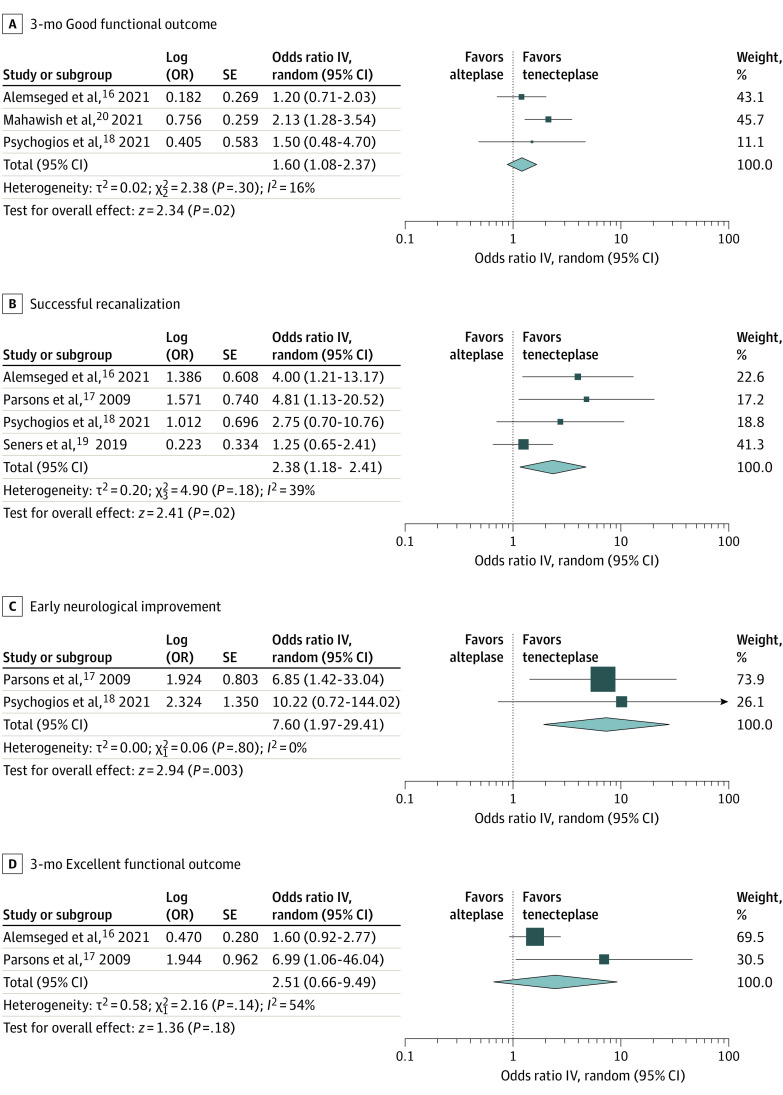
Adjusted Analyses on the Comparison Between Intravenous Tenecteplase and Alteplase for the Outcomes of 3-Month Good Functional Outcome (Modified Rankin Scale 0-2), Successful Recanalization, Early Neurological Improvement, and 3-Month Excellent Functional Outcome (Modified Rankin Scale 0-1) The size of squares is proportional to the weight of each study. Horizontal lines indicate the 95% CI of each study; diamond, the pooled estimate with 95% CI.

Unadjusted and adjusted analyses for primary and secondary outcomes of interest are briefly summarized in Table 2. Patients receiving tenecteplase had higher odds of 3-month good functional outcome with crude OR (1.22; 95% CI, 0.90-1.66) (Figure 2A) and adjusted OR (1.60; 95% CI, 1.08-2.37) (Figure 3A), successful recanalization with crude OR (2.82; 95% CI, 1.12-7.10) (Figure 2B) and adjusted OR (2.38; 95% CI, 1.18-4.81) (Figure 3B), and early neurologic improvement with crude OR (4.88; 95% CI, 2.03-11.71) (Figure 2C) and adjusted OR (7.60; 95% CI, 1.97-29.41) (Figure 3C). No significant differences were detected in the probability of 3-month excellent functional outcome with crude OR (1.53; 95% CI, 0.81-2.91) (Figure 2D) and adjusted OR (2.51, 95% CI, 0.66-9.49) (Figure 3D); the risk difference between intravenous tenecteplase and intravenous alteplase in the pooled crude analysis was 10% with the lower bound of the 95% CI (−5% to 26%) for treatment effect difference at −5%. In addition, no significant differences were detected in the probability of 3-month symptomatic intracranial hemorrhage crude OR (0.97; 95% CI, 0.44-2.16) (eFigure 1A in the Supplement) and adjusted OR (1.16; 95% CI, 0.13-10.50) (eFigure 2 in the Supplement), or any parenchymal hematoma crude OR (1.20; 95% CI, 0.24-5.95) (eFigure 1B in the Supplement). There was little heterogeneity in the results provided from included studies, except for the crude association of treatment with successful recanalization (*I*^2^ = 65%), the adjusted associations of treatment with the likelihood of excellent functional outcome (*I*^2^ = 54%) and symptomatic intracranial hemorrhage (*I*^2^ = 69%). Evidence of publication bias (small-study effect) was noted in the funnel plot of the crude (eFigure 3A in the Supplement) but not of the adjusted association (eFigure 3B in the Supplement) of treatment with the primary outcome of interest.

**Table 2.** Associations Between Intravenous Tenecteplase vs Alteplase and Prespecified Outcomes

Outcome	No. studies	OR (95% CI)	*I*^2^, %	*P* value for Cochran Q
**Crude**				
3-mo good functional outcome (mRS 0-2)	5^a^	1.22 (0.90-1.66)	16	.03
Successful recanalization	4	2.82 (1.12-7.10)	65	.04
Early neurologic improvement	2	4.88 (2.03-11.71)	0	.35
3-mo excellent functional outcome (mRS 0-1)	3	1.53 (0.81-2.91)	0	.42
Symptomatic intracranial hemorrhage	5	0.97 (0.44-2.16)	15	.32
Parenchymal hematoma	3	1.20 (0.24-5.95)	26	.26
**Adjusted**				
3-mo good functional outcome (mRS 0-2)	3	1.60 (1.08-2.37)	16	.30
Successful recanalization	4	2.38 (1.18-4.81)	39	.18
Early neurologic improvement	2	7.60 (1.97-29.41)	0	.80
3-mo excellent functional outcome (mRS 0-1)	2	2.51 (0.66-9.49)	54	.14
Symptomatic intracranial hemorrhage	2	1.16 (0.13-10.50)	69	.07
Parenchymal hematoma	NA	NA	NA	NA

Abbreviations: mRS, modified Rankin Scale; NA, not available; OR, odds ratio.

^a^ Data on the primary outcome of interest were not available.^21^

## Discussion

To our knowledge, the present study is the first meta-analysis of nonrandomized evidence on the comparison between tenecteplase and alteplase in the treatment of AIS using data from different clinical settings. The results are similar to a meta-analysis of RCT data on this same issue.^22^ Tenecteplase use as an intravenous thrombolytic agent for patients with AIS (with and without an underlying large-vessel occlusion) was associated with higher odds of early neurologic improvement and good functional outcome compared with intravenous alteplase. Tenecteplase administration was also associated with a 2-fold higher likelihood of successful recanalization in patients with acute intracranial vessel occlusions compared with intravenous alteplase. No significant difference in the risk of intracranial bleeding between the 2 intravenous thrombolytic agents was noted.

Our findings are in line with evidence from a systematic review and meta-analysis of RCTs,^3^ suggesting that patients with confirmed large-vessel occlusions receiving intravenous tenecteplase have a 3-fold higher odds of successful recanalization (OR, 3.05; 95% CI, 1.73-5.40) and 2-fold higher odds of favorable functional outcome (mRS score, 0-2) at 3 months (OR, 2.06; 95% CI, 1.15-3.69), with no significant increase in the risk of intracranial bleeding, compared with those receiving intravenous alteplase. Consistent with our findings, to our knowledge, neither individual RCTs nor other meta-analyses published to date have suggested any safety concerns with the use of tenecteplase compared with alteplase on the risks of intracranial hemorrhage and all-cause mortality.^3,22^

Tenecteplase seems to achieve fast recanalization in patients with large-vessel occlusion at a consistent rate of 20%, which is independent of whether the patients are secondarily transferred or directly admitted to a center capable of performing endovascular procedures.^23^ This analysis also shows that intravenous tenecteplase treatment was associated with an increased likelihood of early neurologic improvement in both crude and adjusted analyses—an association that has been reported in a previous meta-analysis of RCTs.^24^ Compelling evidence for tenecteplase superiority as a thrombolytic agent compared with alteplase has been provided to date only for patients with confirmed large-vessel occlusions.^25,26^ Studies that have included all patients presenting with symptoms suggestive of AIS did not prove the superiority of tenecteplase over alteplase.^27,28^ This disparity on the outcomes associated with tenecteplase in different study populations has been reflected in the current guidelines from the European Stroke Organization^5^ and American Heart Association/American Stroke Association^6^ suggesting that tenecteplase treatment may be considered only for patients with confirmed acute large-vessel occlusion who are eligible for both intravenous thrombolysis and subsequent endovascular thrombectomy.

In the present meta-analysis, no difference in the likelihood of 3-month excellent functional outcome was detected between patients receiving intravenous tenecteplase or intravenous alteplase. However, in our analysis, the risk difference between intravenous tenecteplase and intravenous alteplase in the pooled crude analysis was 10% with the lower bound of the 95% CI (−5% to 26%) for treatment effect difference at −5%. This noninferiority margin of −5% for treatment effect difference has been suggested as the minimal clinically important difference for acute stroke therapies by a previous survey of stroke experts and used in a previous meta-analysis comparing intravenous tenecteplase with intravenous alteplase for the treatment of AIS within the setting of RCTs.^22^

### Limitations

This study has limitations. First, included studies were nonrandomized; thus, imbalances in patient characteristics are expected between the intravenous tenecteplase and intravenous alteplase groups. For this reason, we also report the adjusted treatment associations with the outcomes of interest. Most of the studies provided adjusted associations for the outcomes of interest using multivariable regression models, with confounders selected either a priori^18,20^ or from univariable associations.^17^ In one study, the process for the selection of covariates included in the multivariable analysis was not provided.^16^ Another study used propensity score matching to address imbalances in baseline characteristics,^19^ using a priori confounders to estimate the propensity score for each patient (eTable 3 in the Supplement). Despite the differences in the methods used for adjustment and the selection process for confounders between included studies, no evidence of heterogeneity was evident in the adjusted analyses for the efficacy end points (Table 2). However, the number of studies included in the adjusted analyses is low because some of the eligible studies provided only crude associations for the outcomes of interest. Second, as highlighted in our bias assessment, the selection of the overall population and alteplase control groups in some of the studies raises concerns about unmeasured confounding owing to the different time periods and institutions in which patients in the case and control groups were treated. Third, although in all studies the standard intravenous alteplase dose (0.9 mg/kg) was used, the intravenous tenecteplase dose varied both within and between studies, ranging from 0.1 to 0.4 mg/kg, with most studies using the 0.25-mg/kg dose (Table 1). In patients with AIS due to a large-vessel occlusion, no significant differences in clinical and radiologic end points were documented between the 0.40- and 0.25-mg/kg doses, with the exception of a numerically higher intracranial bleeding risk with the 0.4-mg/kg dose.^29^ Fourth, there was no central adjudication of the symptomatic intracranial bleeding and successful recanalization events or blinding of the clinical outcome assessors for the majority of included studies.

## Conclusions

This meta-analysis provides supporting evidence from nonrandomized studies that intravenous tenecteplase may be a treatment option for patients with AIS that is associated with more favorable clinical outcomes compared with intravenous alteplase. This hypothesis is being evaluated in ongoing large RCTs examining the utility of intravenous tenecteplase for the treatment of patients with AIS presenting within 4.5 hours^30,31,32,33,34^ or 24 hours^35,36^ from symptom onset and in patients presenting after 4.5 hours from stroke onset or with unknown onset time.^37,38,39,40^ Based on the comparable safety profile of both thrombolytic agents, supported by both RCT and nonrandomized evidence, enrollment in the aforementioned ongoing RCTs appears to be appropriate.

## Notes

Supplement. **eTable 1.** Definition of Successful Recanalization in Patients With Confirmed Intracranial Vessel Occlusion Across Included Studies **eTable 2.** Definition of Early Neurological Improvement at 24h Across Included Studies **eTable 3.** Adjustment for Potential Confounders Across Included Studies **eTable 4.** Quality Assessment of Included Studies With the Newcastle-Ottawa Scale **eFigure 1.** Unadjusted Analyses on the Comparison Between Tenecteplase and Alteplase for the Outcomes of (A) Symptomatic Intracranial Hemorrhage and (B) Parenchymal Hematoma Following Intravenous Thrombolysis **eFigure 2.** Adjusted Analyses on the Comparison Between Tenecteplase and Alteplase for the Outcomes of Symptomatic Intracranial Hemorrhage Following Intravenous Thrombolysis **eFigure 3.** Funnel Plots on the Unadjusted and Adjusted Comparison Between Intravenous Tenecteplase and Alteplase for the Primary Outcome of Interest (Three-Month Good Functional Outcome; Modified Rankin Scale 0-2)
