# Supplementary material for: Off-Label Use of Tenecteplase for the Treatment of Acute Ischemic Stroke: A Systematic Review and Meta-analysis
Source: JAMA Netw Open. 2022 Mar 31;5(3):e224506. doi: 10.1001/jamanetworkopen.2022.4506 (PMC8972028; doi:10.1001/jamanetworkopen.2022.4506)
Supplement: Supplement. — eTable 1. Definition of Successful Recanalization in Patients With Confirmed Intracranial Vessel Occlusion Across Included Studies eTable 2. Definition of Early Neurological Improvement at 24h Across Included Studies eTable 3. Adjustment for Potential Confounders Across Included Studies eTable 4. Quality Assessment of Included Studies With the Newcastle-Ottawa Scale eFigure 1. Unadjusted Analyses on the Comparison Between Tenecteplase and Alteplase for the Outcomes of (A) Symptomatic Intracranial Hemorrhage and (B) Parenchymal Hematoma Following Intravenous Thrombolysis eFigure 2. Adjusted Analyses on the Comparison Between Tenecteplase and Alteplase for the Outcomes of Symptomatic Intracranial Hemorrhage Following Intravenous Thrombolysis eFigure 3. Funnel Plots on the Unadjusted and Adjusted Comparison Between Intravenous Tenecteplase and Alteplase for the Primary Outcome of Interest (Three-Month Good Functional Outcome; Modified Rankin Scale 0-2) [file jamanetwopen-e224506-s001.pdf]

Supplementary Online Content

Katsanos AH, Psychogios K, Turc G, et al. Off-label use of tenecteplase for the treatment of acute ischemic stroke: a systematic review and meta-analysis. *JAMA Netw Open*. 2022;5(3):e224506. doi:10.1001/jamanetworkopen.2022.4506

eTable 1. Definition of Successful Recanalization in Patients With Confirmed Intracranial Vessel Occlusion Across Included Studies

eTable 2. Definition of Early Neurological Improvement at 24h Across Included Studies

eTable 3. Adjustment for Potential Confounders Across Included Studies

eTable 4. Quality Assessment of Included Studies With the Newcastle-Ottawa Scale

eFigure 1. Unadjusted Analyses on the Comparison Between Tenecteplase and Alteplase for the Outcomes of (A) Symptomatic Intracranial Hemorrhage and (B) Parenchymal Hematoma Following Intravenous Thrombolysis

eFigure 2. Adjusted Analyses on the Comparison Between Tenecteplase and Alteplase for the Outcomes of Symptomatic Intracranial Hemorrhage Following Intravenous Thrombolysis

eFigure 3. Funnel Plots on the Unadjusted and Adjusted Comparison Between Intravenous Tenecteplase and Alteplase for the Primary Outcome of Interest (Three-Month Good Functional Outcome; Modified Rankin Scale 0-2)

This supplementary material has been provided by the authors to give readers additional information about their work.

© 2022 Katsanos AH et al. *JAMA Network Open.*

eTable 1: Definition of successful recanalization in patients with confirmed intracranial vessel occlusion across included studies.

Study name	Definition
Alemseged et al,[1] 2021	mTICI 2b–3 on initial angiogram (before endovascular therapy was performed) or reperfusion ≥50% of the involved territory on multimodal imaging
Parsons et al,[2] 2009	24-hour CTA or MRA demonstrating partial flow or normal flow using adapted Thrombolysis in Myocardial Infarction (TIMI) criteria (TIMI≥2)
Psychogios et al,[3] 2021	24-hour CTA or MRA or Transcranial Doppler demonstrating normal flow
Seners et al,[4] 2019	mTICI 2b-3 on pre-mechanical thrombectomy first angiographic run or non-invasive vascular imaging
Mahawish et al,[5] 2021	N/A
Warrach et al,[6] 2021	N/A

mTICI: modified Thrombolysis in Cerebral Infarction; CTA: computed tomography angiography; MRA: magnetic resonance angiography; TIMI: Thrombolysis in Myocardial Infarction; N/A: not available

© 2022 Katsanos AH et al. *JAMA Network Open.*

eTable 2: Definition of early neurological improvement at 24h across included studies.

Study name	Definition
Alemseged et al,[1] 2021	N/A
Parsons et al,[2] 2009	Reduction in the baseline NIHSS score of at least 8 points
Psychogios et al,[3] 2021	Reduction in the baseline NIHSS score of at least 8 points
Seners et al,[4] 2019	N/A
Mahawish et al,[5] 2021	N/A
Warrach et al,[6] 2021	N/A

NIHSS: National Institutes of Health Stroke Scale; N/A: not available

© 2022 Katsanos AH et al. *JAMA Network Open.*

eTable 3: Adjustment for potential confounders across included studies

Study name	Adjusted variables	Method for adjustment
Alemseged et al,[1] 2021	Age, NIHSS score, needle-to-arterial puncture time, cardioembolic etiology	Multivariable regression (selection process for confounders not described)
Parsons et al,[2] 2009	Age, NIHSS, serum glucose, infarct core volume, penumbra volume, percent penumbra, TIMI grade 0 (baseline)	Multivariable regression (confounders selected based on p-values from univariable analyses)
Psychogios et al,[3] 2021	Age, baseline NIHSS score, presence of proximal intracranial occlusion	Multivariable regression (a priori defined confounders)
Seners et al,[4] 2019	Age, NIHSS, onset-to-IVT time, occlusion site, thrombus length	Propensity score matching (a priori defined confounders)
Mahawish et al,[5] 2021	Age, sex, pre-morbid mRS, onset-to-door-time, NIHSS, thrombectomy, door-to-needle time	Multivariable regression (a priori defined confounders)
Warrach et al,[6] 2021	N/A	N/A

NIHSS: National Institutes of Health Stroke Scale; IVT: intravenous thrombolysis; mRS: modified Rankin Scale; TIMI: Thrombolysis in Myocardial Infarction, N/A: not available

© 2022 Katsanos AH et al. *JAMA Network Open.*

eTable 4: Quality assessment of included studies with the Newcastle–Ottawa Scale.

Study name	Selection	Comparability	Outcome	Overall score
Alemseged et al,[1] 2021	****	**	***	9/9
Parsons et al,[2] 2009	***	*	***	7/9
Psychogios et al,[3] 2021	***	**	**	7/9
Seners et al,[4] 2019	***	**	**	7/9
Mahawish et al,[5] 2021	***	**	**	7/9
Warrach et al,[6] 2021	N/A	N/A	N/A	N/A
Overall Score	16/20	9/10	12/15	37/45

N/A: not available

© 2022 Katsanos AH et al. *JAMA Network Open.*

eReferences:

1. Alemseged F, Ng FC, Williams C, et al. Tenecteplase vs Alteplase Before Endovascular Therapy in Basilar Artery Occlusion. Neurology. 2021;96:e1272-e1277.

2. Parsons MW, Miteff F, Bateman GA, et al. Acute ischemic stroke: imaging-guided tenecteplase treatment in an extended time window. Neurology. 2009;72:915-21.

3. Psychogios K, Palaiodimou L, Katsanos AH, et al. Real-world comparative safety and efficacy of tenecteplase versus alteplase in acute ischemic stroke patients with large vessel occlusion. Ther Adv Neurol Disord. 2021;14:1756286420986727.

4. Seners P, Caroff J, Chausson N, et al. Recanalization before Thrombectomy in Tenecteplase vs. Alteplase-Treated Drip-and-Ship Patients. J Stroke. 2019;21:105-107.

5. Mahawish K, Gommans J, Kleinig T, Lallu B, Tyson A, Ranta A. Switching to Tenecteplase for Stroke Thrombolysis: Real-World Experience and Outcomes in a Regional Stroke Network. Stroke. 2021;52:e590-e593.

6. Warach SJ, Miley JT, Mawla M, et al. Abstract P2: Results of a Prospective Observational Cohort Study of Tenecteplase as Standard of Care Stroke Thrombolytic. Stroke. 2021;52:AP2. https://doi.org/10.1161/str.52.suppl_1.P2

© 2022 Katsanos AH et al. *JAMA Network Open.*

eFigure 1: Unadjusted analyses on the comparison between tenecteplase and alteplase for the outcomes of (A) symptomatic intracranial hemorrhage and (B) parenchymal hematoma following intravenous thrombolysis.

A.

Study or Subgroup	TNK Events	TNK Total	alteplase Events	alteplase Total	Weight	Odds Ratio IV, Random, 95% CI
Alemseged et al, 2021	1	19	1	91	7.6%	5.00 [0.30, 83.69]
Mahawish et al, 2021	5	283	19	555	43.0%	0.51 [0.19, 1.37]
Parsons et al, 2009	0	15	1	35	5.8%	0.74 [0.03, 19.25]
Psychogios et al, 2021	3	19	2	39	15.8%	3.47 [0.53, 22.80]
Warach et al, 2021	3	151	8	351	27.9%	0.87 [0.23, 3.32]
**Total (95% CI)**		**487**		**1071**	**100.0%**	**0.97 [0.44, 2.16]**
Total events	12		31			

Heterogeneity: Tau² = 0.13; Chi² = 4.68, df = 4 (P = 0.32); I² = 15%
Test for overall effect: Z = 0.07 (P = 0.94)

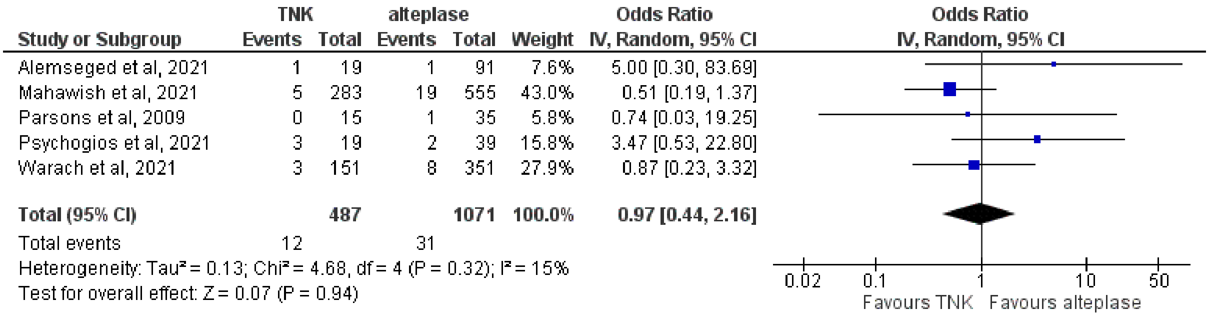

B.

Study or Subgroup	TNK Events	TNK Total	alteplase Events	alteplase Total	Weight	Odds Ratio IV, Random, 95% CI
Alemseged et al	0	19	3	91	22.9%	0.65 [0.03, 13.07]
Parsons et al	0	15	4	35	23.2%	0.23 [0.01, 4.47]
Psychogios et al	4	19	3	39	53.9%	3.20 [0.64, 16.07]
**Total (95% CI)**		**53**		**165**	**100.0%**	**1.20 [0.24, 5.95]**
Total events	4		10			

Heterogeneity: Tau² = 0.56; Chi² = 2.69, df = 2 (P = 0.26); I² = 26%
Test for overall effect: Z = 0.22 (P = 0.82)

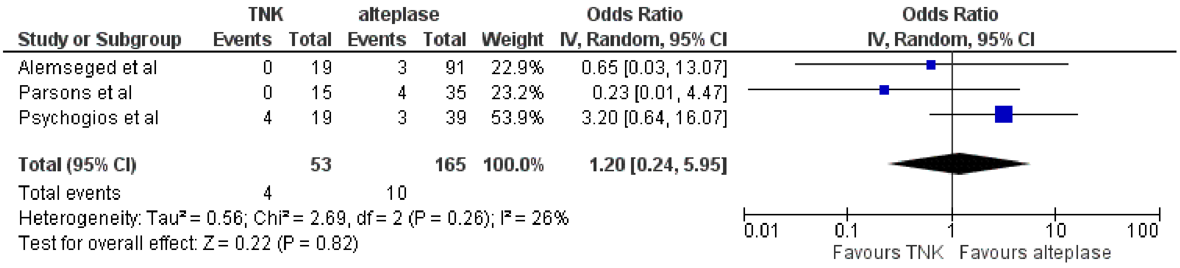

© 2022 Katsanos AH et al. *JAMA Network Open.*

eFigure 2: Adjusted analyses on the comparison between tenecteplase and alteplase for the outcomes of symptomatic intracranial hemorrhage following intravenous thrombolysis.

.

Study or Subgroup	log[Odds Ratio]	SE	Weight	Odds Ratio IV, Random, 95% CI
Mahawish et al, 2021	-0.821	0.656	57.1%	0.44 [0.12, 1.59]
Psychogios et al, 2021	1.447	1.079	42.9%	4.25 [0.51, 35.23]
**Total (95% CI)**			**100.0%**	**1.16 [0.13, 10.50]**

Heterogeneity: Tau² = 1.77; Chi² = 3.23, df = 1 (P = 0.07); I² = 69%
Test for overall effect: Z = 0.13 (P = 0.89)

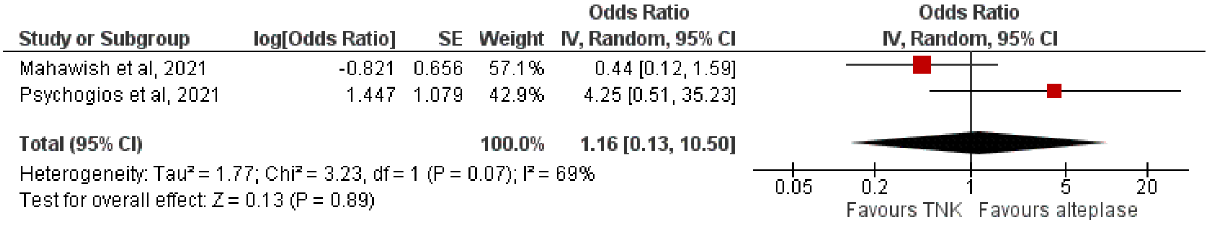

© 2022 Katsanos AH et al. *JAMA Network Open.*

eFigure 3: Funnel plots on the (A) unadjusted and (B) adjusted comparison between intravenous tenecteplase and alteplase for the primary outcome of interest (three-month good functional outcome; modified Rankin Scale 0-2)

A.

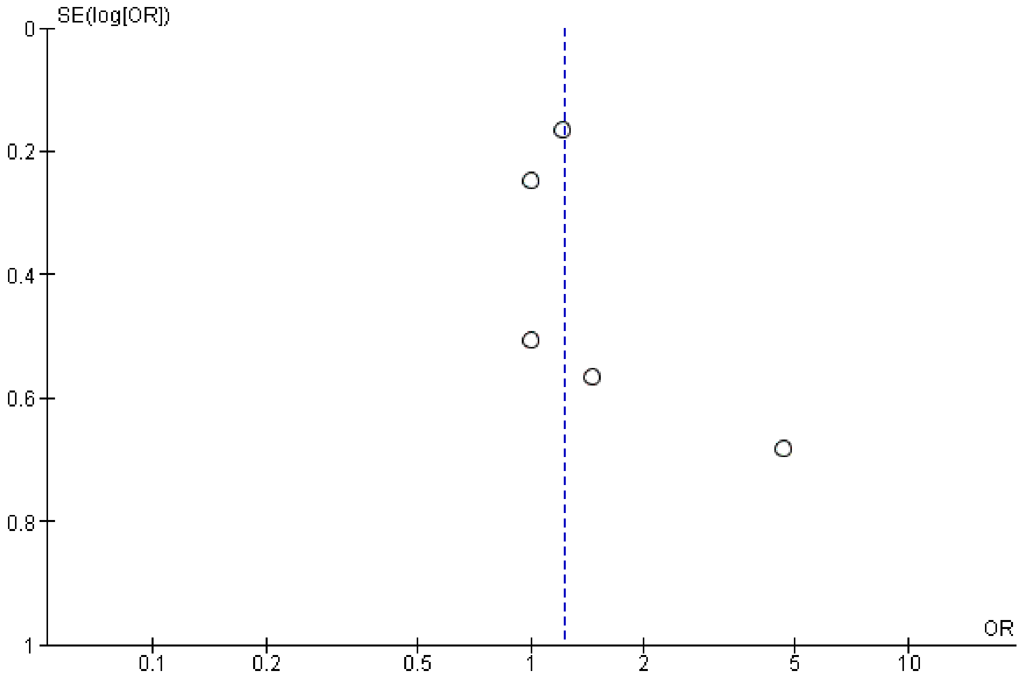

B.

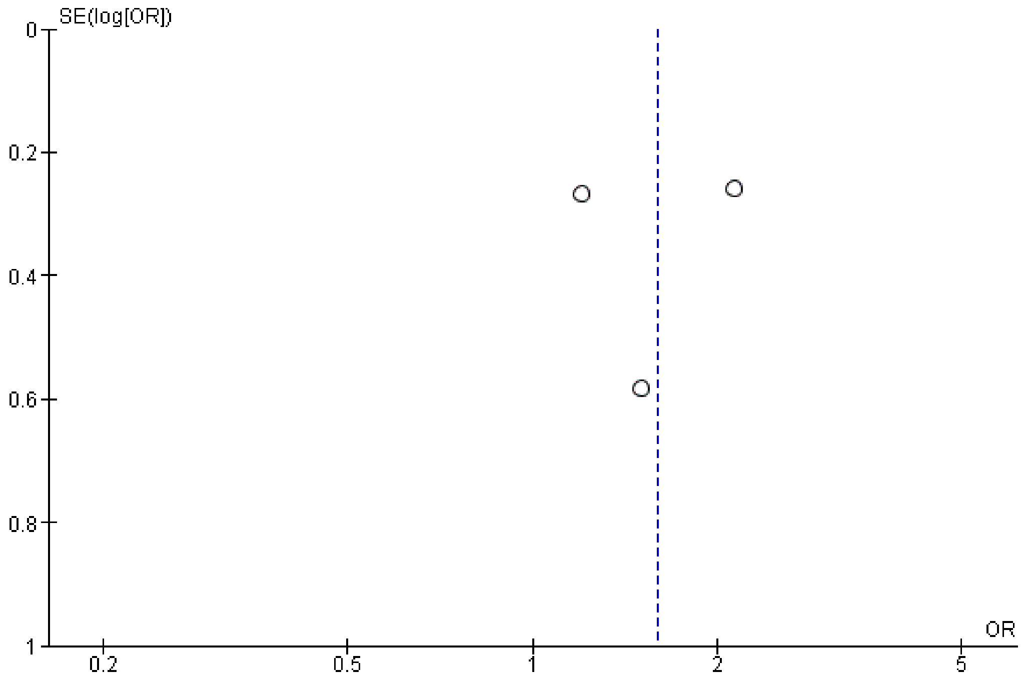

© 2022 Katsanos AH et al. *JAMA Network Open.*
